# Building resilient food safety systems through a One Health approach to prepare for the next pandemic

**DOI:** 10.1038/s41538-026-00798-4

**Published:** 2026-07-01

**Authors:** Maura Di Martino, Sean Shadomy, Diletta Topazio, Jeffrey T. LeJeune

**Affiliations:** Agrifood Systems and Food Safety Division at the Food and Agriculture Organization of the United Nations (FAO), Rome, Italy

**Keywords:** Health care, Risk factors

## Abstract

Pandemics threaten the agrifood systems on which societies depend, yet their effects on these critical systems receive little attention. This review examines the relationship between pandemics and food through a One Health lens, from their origins out of interactions between humans, animals, and the environment to their impact on food safety and agrifood systems. It then proposes actions to strengthen the preparedness of these systems to defend against these threats.

## Introduction

Pandemics threaten not only human health directly but also the safety, stability, and resilience of the agrifood systems on which societies depend. While the clinical and epidemiological impacts of pandemics are well studied, their implications for food safety, including regulation, oversight, and consumer protection, have received comparatively limited attention.

Importantly, food safety risks arise in all pandemics, regardless of whether the causative pathogen is foodborne. Even when transmission does not occur through food, public health measures such as lockdowns, market closures, and movement restrictions, workforce reductions due to illness, and the diversion of laboratory resources, can disrupt food safety operations, undermining inspection and regulatory oversight^1–5^. The COVID-19 pandemic exemplified these systemic vulnerabilities. Public health and regulatory disruptions included reduced inspection frequencies, the repurposing of food testing laboratories for emergency diagnostics, delays in traceability and recall activities, and increased reliance on informal or unregulated food supply channels^6^. Together, these factors created conditions more conducive to food contamination and fraud.

Although SARS-CoV-2 infections have not been linked to consumption of food^7,8^, the diversion of resources and attention toward pandemic response reduced the capacity to enforce routine food hygiene and traceability standards. At the same time, foodborne pathogens themselves can cause pandemics or large-scale public health emergencies. For instance, *Vibrio cholerae*, the causative agent of cholera, is transmitted through fecal-contaminated food and water and has caused seven major pandemics since the 19th century, with outbreaks still affecting millions of people worldwide, where water and sanitation infrastructure are inadequate^9,10^. Additionally, antimicrobial-resistant (AMR) foodborne pathogens pose an emerging pandemic risk, as resistant infections are becoming increasingly difficult to treat and control worldwide^11^.

The One Health approach has made critical progress in upstream zoonotic disease surveillance and wildlife trade regulation^12^, yet its application has remained largely focused on disease emergence, with limited attention to downstream effects on food safety governance and operational resilience^13^. This is a critical oversight given that pandemics can rapidly permeate agrifood systems, affecting processing, distribution, trade, the stability of regulatory systems, and consumer behavior^14^.

This narrative review draws on peer-reviewed literature and reports from international organizations published between 1996 and 2025, identified through targeted searches of bibliographic databases and institutional repositories. It examines the relationship between pandemics and food safety through a One Health lens. It first outlines the origins of pandemics across human, animal, and environmental systems, then considers how such events affect food safety, governance, institutional oversight, and consumer protection. Drawing on past and present outbreaks, it argues that food safety, often relegated to a downstream issue, is in fact central to public health resilience, agrifood system stability, and public trust during health crises. At the same time, this review recognizes that food safety alone is a necessary but not sufficient condition for food security, as access, availability, affordability, infrastructure, and broader socio-economic factors remain critical determinants of food security outcomes^13,15^. Finally, this review proposes steps that countries and regions can take to address food safety within pandemic preparedness as essential actions to protecting populations and preserving confidence in global agrifood systems during crises.

### Pandemic emergence, agrifood systems and One Health

Pandemic emergence is driven by a complex interplay of biological, environmental, and socio-economic factors. Zoonotic diseases comprise the majority of both known human infectious diseases and newly emerging ones^16,17^. Spillover events frequently occur where agrifood systems interface with natural ecosystems, locations where humans, domesticated animals, and wildlife interact closely. While improvements in animal husbandry, food handling, and hygiene have reduced spillover risks, emerging challenges such as rapid urbanization, agricultural intensification, biodiversity loss, and globalized food supply chains are increasing the frequency of these interactions^1,18,19^.

**Fig. 1:**
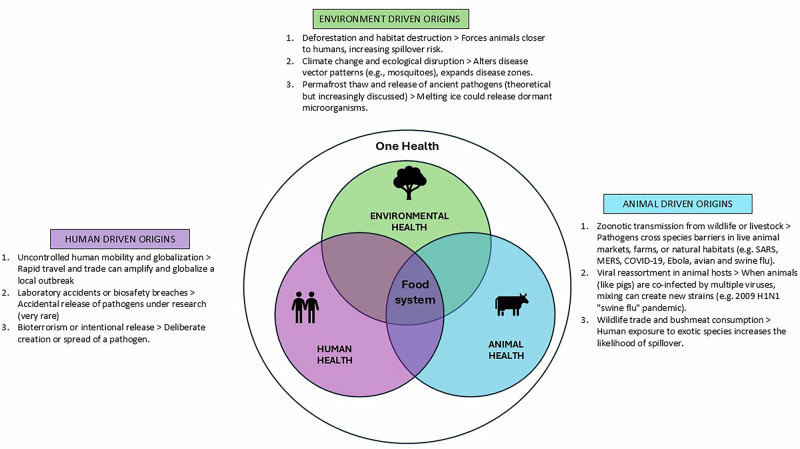
One Health sector drivers of pandemics in agrifood systems. The Venn diagram illustrates the interconnected domains of Human Health, Animal Health, and Environmental Health, with the Food System at their intersection. Each domain contributes distinct but overlapping drivers of pandemic emergence: human-driven, animal-driven, and environment-driven. These drivers interact within agrifood systems, influencing the risk, scale, and transmission pathways of emerging infectious diseases.

Figure 1 illustrates the interconnected drivers of pandemics within a One Health framework, categorizing them into three overlapping domains: human-driven, animal-driven, and environment-driven. These drivers not only facilitate disease emergence and spread but can also introduce complex food safety risks across the supply chain. For instance, agricultural expansion (environment-driven) can bring humans and their livestock into closer contact with wildlife (animal-driven), increasing the likelihood of novel pathogens entering food production systems. Meanwhile, global mobility and trade (human-driven) can rapidly accelerate pathogen dissemination and the cross-border movement of contaminated food products.

Crucially, localized spillovers can escalate into epidemics and, under the right conditions, pandemics. Factors such as population density, global connectivity, weak surveillance systems, and delayed responses contribute to rapid disease amplification. In such contexts, agrifood systems can play an important role in potentially exacerbating the spread of a pathogen through extensive distribution networks or due to inadequate biosecurity and can influence the routes and scale of transmission.

Most foodborne illnesses occur as localized outbreaks, which are usually contained through established food safety regulations, traceability systems, and public health interventions, even when international trade is involved. Historically, prompt identification and control of contaminated food or water sources have proven effective in stopping outbreaks, even when the causative pathogen was not yet known. A notable example is John Snow’s 1854 cholera investigation in London, where removing access to a contaminated water pump successfully halted transmission, decades before *Vibrio cholerae* was identified^20^. Today, improved tools and guidance enable rapid detection and response to foodborne events, preventing their escalation into wider public health crises^21^.

However, the possibility of a foodborne pathogen contributing to a future pandemic cannot be excluded. For such a situation to arise, two conditions are necessary: the pathogen must be capable of infecting humans through handling or consumption of contaminated food, and it must additionally have or acquire the ability for efficient human-to-human transmission making containment more challenging, as seen in explosive outbreaks of norovirus which can spread via contaminated food and drink, and by direct contact with infected persons and contaminated surfaces. Mutations that increase pathogen stability in food, prolong shedding, or enhance environmental persistence could also amplify their potential impact. Hence, agrifood systems can act as spillover points or amplifiers, and vigilant monitoring and preparedness are essential to anticipate and respond to emerging threats^22^.

Given these dynamics, there is growing recognition that pandemic preparedness must address not only upstream drivers, such as through wildlife surveillance and animal trade biosecurity, but also the structure and governance of agrifood systems.^1,19^.

### Pandemic drivers in agrifood systems

Pandemic emergence is also shaped by complex interactions between ecological, geographic, socio-economic, and agrifood system dynamics. Regions characterized by high wildlife and ecosystem diversity, dense human populations, and rapid land-use change are recognized hotspots for zoonotic spillover, particularly where agrifood systems interface with wildlife habitats^17,23^. Climate change amplifies these risks by intensifying extreme weather events that bring wildlife, livestock, and humans into closer contact and altering disease ecologies, increasing pathogen survival and replication^18,24^, and expanding the range of vectors such as *Aedes* mosquitoes^25,26^. For example, tick-borne encephalitis cases and the number of affected countries in Europe have increased over the past decade, as *Ixodes ricinus* tick vector development has accelerated, its activity has extended with milder, shorter winters, and its distribution expands to higher altitudes and previously unaffected regions^27,28^.

Global trade of food can transport pathogens far from their origin. Cholera outbreaks in cholera-free countries have been linked to imported contaminated foods^29–31^. Moreover, the persistent storage of food in sub-zero temperatures throughout complex supply chains preserves not only food but also maintains pathogen infectivity. For example, frozen berries have emerged as one of the most common vehicles for foodborne viral outbreaks, particularly norovirus and hepatitis A^32^. Additionally, inadequate or uneven cold-chain infrastructure, particularly in low-resource or remote regions, increases the risk of pathogen survival and amplification of bacteria in perishable foods, undermining food safety controls^33^.

Rising demand for animal-source foods has led to increased meat production and increasing global trade in live animals and meat and dairy products^34^. This trade can facilitate the long-distance movement of pathogens and introduction into new geographic regions and new host species, such as through live animal markets, increasing the risk for pathogen adaptation and the emergence of new strains. The cross-border trade and crowding of farmed and captive wildlife in these markets facilitate interspecies transmission, as suspected in the emergence of SARS-CoV and SARS-CoV-2^35,36^. Furthermore, these market environments serve as high-risk interfaces for zoonotic spillover into humans, as seen with avian influenza and MERS-CoV outbreaks^36,37^. Trade in wild meat poses an additional risk for zoonotic pathogen introduction into new regions^38^.

Modern food production systems such as intensive animal farming foster the evolution and amplification of zoonotic pathogens^39^. Live animal importation to improve genetics followed by viral reassortment in swine, for example, led to the emergence of the 2009 H1N1 pandemic influenza strain^40^. Similarly, the successive emergence of multidrug-resistant Salmonella strains may be linked to animal production practices^41,42^. The evolution of avian influenza H5N1 highlights this potential role of agrifood systems in disease emergence. The spread of clade 2.3.4.4b across bird and mammal populations demonstrates the virus’ cross-species adaptation^43,44^. A single hemagglutinin (HA) protein amino acid change in a human isolate of clade 2.3.4.4b circulating in dairy cattle may facilitate human-type receptor binding, raising concerns for potential human transmissibility^45^. The virus’s persistence in raw dairy products further highlights its potential for foodborne transmission^46–48^.

**Fig. 2:**
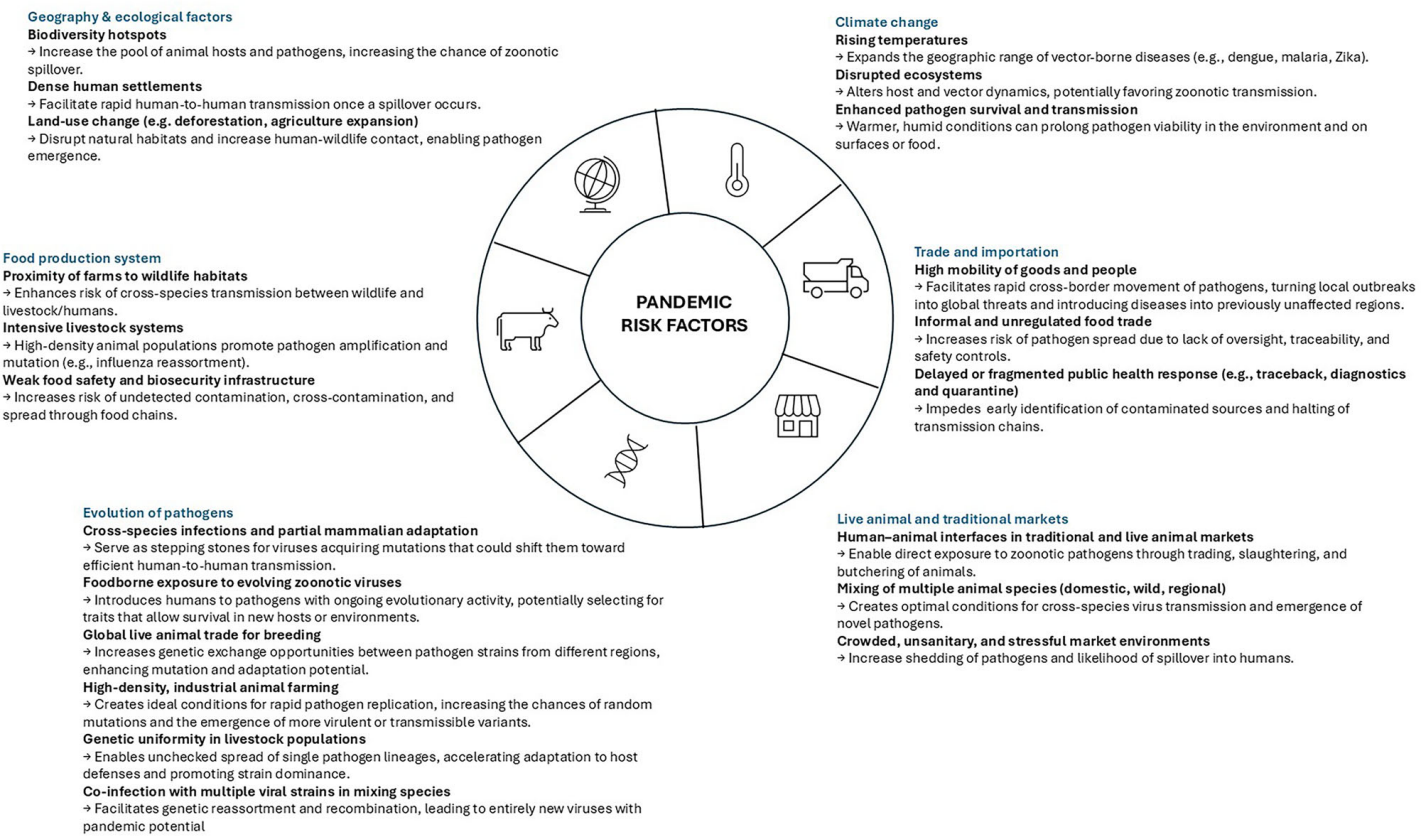
Agrifood systems factors contributing to the spread of pathogens globally. The diagram illustrates six interconnected domains contributing to pandemic risk: geography and ecological factors, climate change, food production systems, trade and importation, pathogen evolution, and socio-political conditions. Each domain highlights specific risk-enhancing mechanisms that facilitate zoonotic spillover, pathogen amplification, and cross-border transmission.

As illustrated in Fig. 2, these drivers converge and interact in ways that compound pandemic risk. Urban density, global travel, live animal markets, intensive agriculture, and climate stress collectively create conditions conducive to pathogen emergence, amplification, and rapid spread. For example, in the Middle East and North Africa, pandemic risks are heightened by conflicts that disrupt health systems, changing land and water use, population movement, and mass gatherings, climate-related impacts, diverse animal husbandry practices and trade routes, suboptimal healthcare and surveillance infrastructure and high antimicrobial resistance burden^49^.

This complex, interconnected landscape underscores the need to strengthen agrifood system resilience, enhance farm-to-fork surveillance, and address upstream drivers, key components of effective pandemic preparedness and global health protection.

### Pandemic risks through the lens of food

Food and food production, processing, and distribution systems provide several pathways through which pathogens with pandemic potential can be introduced into human populations, and which can influence the evolution of an outbreak into a Public Health Emergency of International Concern (PHEIC), epidemic or a pandemic through repeated food-related introductions^3^. These pathways are not mutually exclusive and include:

(1) exposure through consumption of infected or contaminated foods;

(2) exposure through contact with or handling of infected food animals, meat and animal products, including in live animal markets and during slaughtering, processing and butchering; and,

(3) exposure through contact and handling of contaminated foods or food packaging.

The 2024 World Health Organization R&D Blueprint for Epidemics identified five bacterial pathogens and 30 viruses representing 11 viral families as posing high risk for causing a PHEIC or pandemic out of 1652 pathogens evaluated, based on transmission patterns, virulence, and countermeasures availability^50^. Supplementary Table 1 includes a proposed framework based on expert opinion and current literature for considering the potential for each of these pathogens to cause such events through food-related exposures, based on the combination of the capacity for introduction through one or more of these pathways and for subsequent propagation in a population via sustained human-to-human transmission or community transmission such as through food or water contamination in settings with poor sanitation or infrastructure breakdown. Of these 35 pathogens, seven (four bacteria and three coronaviruses) have the greatest potential for both food-related introduction and to spread widely through human populations. The ability for bacterial pathogens to proliferate outside of the host, especially in food matrices high in organic and moisture content, increases the likelihood for their introduction or reintroduction due to food handling and preparation practices. They can persist in contaminated raw meat, seafood, dairy, and other products, for instance, following intestinal content contamination of carcasses at slaughter or raising seafood in sewage-contaminated waters, and can proliferate in foods through inadequate cooking and improper storage temperatures and attain sufficient numbers to reach an infective dose^29,42^.

### Mapping pandemic impacts on food safety systems

Beyond their direct impacts on human health, pandemics can destabilize food safety systems by disrupting regulatory continuity, laboratory capacity, market surveillance, and consumer protection^6^. Evidence from the COVID-19 pandemic and earlier public health emergencies demonstrates that these disruptions arise through both direct operational constraints and indirect institutional and behavioral responses^2–4^. To characterize these impacts, it is useful to distinguish between direct and indirect consequences, as outlined in Table 1. Direct effects include diminished inspections, occupational risk, and reduced lab support. Even in countries with relatively robust food safety infrastructures, COVID-19 exposed systemic weaknesses, although typically at a smaller scale than in low-resource settings^14^. Indirect effects stem from institutional responses, such as lockdowns and reduced or deferred regulatory enforcement, which can delay recalls and increase reliance on unregulated markets. As public health emergencies unfold, attention and resources are overwhelmingly redirected toward outbreak control, reducing capacity for inspections and hygiene monitoring, especially in low-resource settings.

**Table 1:** Direct and indirect food safety hazards of pandemics pandemic origin

Domain	Expected effect on the agrifood system	Food safety hazard
Human-driven	Direct	Contamination by infected food handlers (e.g., Hepatitis A, norovirus, Salmonella)
		Breakdown in hygiene during food preparation that allows pathogen amplification
		Cross-contamination in food service settings, e.g., school kitchen and catering outbreaks
		Poor sanitation infrastructure enabling foodborne outbreaks to scale
		Pathogen introduction from poor handling of ready-to-eat foods or raw produce
		Overcrowded food facilities without infection control, exacerbating spread via surfaces or food
	Indirect	Workforce shortages reduce the number of trained food safety inspectors or handlers
		Testing labs repurposed for pathogen diagnostics, limiting capacity for food safety monitoring
		Relaxed regulations and standards during emergencies compromise food controls
		Suspended training or certification programs for food businesses and handlers
		Surge in informal food sales where hygiene is unregulated (e.g., street vendors during lockdowns)
		Weakened traceability systems make food recalls and investigations slower
		Misinformation (e.g., about food transmission of the pandemic pathogen) may lead to unsafe substitutions or hoarding
		Food fraud and adulteration increase when trusted supply chains are disrupted
Environment-driven	Direct	Pathogens in contaminated water, soil, or wild animals enter the food chain (e.g., Vibrio, Leptospira, Cyclospora)
		Toxin-producing fungi or algae (e.g., aflatoxins, ciguatoxins) proliferate under warming or humidity
		Heavy metal accumulation in crops or seafood due to polluted ecosystems (e.g., mercury, cadmium)
		Pests or vectors (e.g., rodents, flies) transfer environmental, zoonotic or fecal-oral pathogens to food
		Permafrost thaw may release ancient pathogens into Arctic agrifood systems
	Indirect	Cold chain breakdowns due to weather, flooding, or energy loss cause spoilage and pathogen growth
		Climate-induced pest outbreaks lead to higher pesticide use and unsafe residues in food
		Water insecurity affects hygiene and sanitation in food preparation and production
		Overburdened testing labs delay detection of contaminants or residues in food
		Regulatory enforcement weakens due to disaster response priorities
		Training disruptions and infrastructure collapse reduce hygiene and safety compliance in agrifood systems
Animal-driven	Direct	Zoonotic pathogen enters the food chain (e.g., Salmonella, Campylobacter, Brucella, avian influenza virus)
		Consumption of contaminated animal products (e.g., undercooked meat, unpasteurized milk)
		Unregulated slaughter and informal animal markets lacking inspection and hygiene
		Inadequate veterinary health monitoring, allowing asymptomatic foodborne transmission
		Antimicrobial-resistant bacteria spread via animal-origin food (e.g., E. coli, MRSA)
		Cross-contamination in slaughterhouses and meat processing facilities
	Indirect	Disruption of veterinary inspections and food chain certification (e.g., movement bans, understaffing)
		Outbreaks in food animal facilities delay safe processing and increase contamination risks
		Illegal or emergency animal trade during border closures bypasses safety controls
		Delayed detection of animal-origin foodborne outbreaks due to overwhelmed systems
		Shortages of animal feed or veterinary drugs may lead to poor animal health and unsafe meat/milk
		Increased informal slaughter or local butchering with no hygiene oversight

Consumer behavior is an integral component of food safety systems, as household food handling, preparation, storage, and sourcing practices directly influence exposure risks and the effectiveness of traceability and consumer protection mechanisms^13^. During pandemics, misinformation and fear of infection may stigmatize certain foods or cultural practices and may lead to unsafe substitutions and increased dependence on informal sources, thereby weakening regulatory oversight and food safety controls, affecting both safety and equity of access. These behavioral responses are shaped by existing socio-economic disparities, which pandemics tend to exacerbate.

At the workforce level, communication barriers, such as limited proficiency in the dominant language of public health messaging, can further undermine risk communication, training, and the effective implementation of food safety and infection prevention measures. These challenges were documented among food processing and meatpacking workers during the COVID-19 pandemic^51^. Workforce-level constraints, in turn, interact with broader structural and infrastructural factors, amplifying pandemic-related pressures on food safety systems. Notably, these behavioral and institutional responses often vary by region, socio-economic context, and position within the food supply chain, rather than aligning strictly along national boundaries^2^.

Mental health pressures compound these challenges. The psychological toll of isolation, job loss, and uncertainty can elevate rates of anxiety, depression, and stress-related behaviors, which may affect hygiene and food safety practices^8^. Disruptions to routines and effective coping mechanisms may result in specific risk-enhancing behaviors, including inadequate cooking of animal-source foods, improper refrigeration or cold-chain maintenance, extended storage of perishable foods, and reduced attention to hygiene during food preparation^14^. At the same time, mental health services and community support systems are often disrupted during pandemics, leaving these secondary food safety risks insufficiently addressed.

Pandemic impacts on food safety systems are shaped by the interaction of regulatory capacity, supply-chain structure, consumer behavior, and regional infrastructure. In practice, these impacts are influenced less by political boundaries than by shared ecological conditions, trade routes, and market linkages, and they vary across different segments of the food supply chain^2,5,13^. Rural and remote communities are especially vulnerable, facing heightened risks due to limited access to regulated markets, constrained cold-chain and distribution infrastructure, and reduced inspection coverage^15^.

Taken together, these dynamics illustrate how pandemics can erode food safety protections at multiple levels of the food system, even when foodborne transmission is not a primary route of disease spread.

### The potential economic impact of a foodborne pandemic

While the COVID-19 pandemic was primarily an airborne health crisis, its global economic fallout provides a valuable reference point for understanding how a pandemic might disrupt international trade and agrifood systems. COVID-19 triggered widespread containment measures that led to a 12.2% decline in global goods trade in Q2 2020 and a 21.4% contraction in services trade compared with the same quarter in 2019^52^. Although these global shocks were severe, most impacts on food trade were indirect. International agrifood supply chains demonstrated relative resilience, as many governments explicitly prioritized maintaining the movement of agricultural commodities across borders to prevent food shortages^3^. However, this resilience was not uniform across contexts. The effects differed by region and by position within the supply chain, with some critical bottlenecks emerging. In particular, the structural divisions between production systems for food destined for home consumption and food supplied to restaurants and institutions result in rigidities in market flow of foods; when demand shifted rapidly toward at-home consumption, packaging systems, processing capacity, and cold-chain infrastructure were in several cases slow or unable to adjust, leading to localized disruptions and uneven impacts across sectors, particularly in regions and supply-chain segments oriented toward food service and institutional markets^53^.

In contrast, a foodborne pandemic would likely generate direct, sector-specific trade disruptions, particularly for perishable goods and globally traded agri-food exports. Such a crisis would be defined by the loss of consumer and regulatory trust in the safety of food products, triggering import bans, precautionary restrictions, and avoidance behavior undermining domestic demand for fresh and minimally processed products and potentially affecting food choices and nutrition. Historical precedents illustrate this dynamic. For example, a 2008 U.S. *Salmonella* outbreak was erroneously first linked to tomatoes, leading to estimated industry losses exceeding $100 million as consumers and retailers rejected tomatoes^54^. Similarly, the 2006 U.S. *E. coli* O157:H7 outbreak from spinach resulted in an estimated $202 million decline in spinach sales in the 68 weeks after FDA issued consumption warnings, plus an additional $60 million loss for other leafy greens^55^. The 2011 European *E. coli* O104:H4 outbreak led to export bans and widespread retail pullbacks^56^. According to the European Commission, these disruptions triggered peak losses of approximately €417 million per week, far exceeding the impact of earlier outbreaks (e.g., 2006 spinach, 2008 *Salmonella*)^57^. Because of prolonged consumer fear and supply-chain disruption, these economic losses likely persisted for 6–8 weeks. Extrapolating these cases to a pandemic context suggests even more severe and widespread trade disruptions. The economic channels of disruption would therefore be different and arguably more asymmetric than those observed during COVID-19.

Countries with export-dependent economies and those deeply integrated into international commodity flows would be disproportionately exposed, particularly where diversification is limited and agrifood exports represent a major source of income. Economies heavily reliant on such exports could experience sudden contractions in export revenues, with potential implications for macroeconomic stability and food security. Domestically, producers would face increased compliance costs associated with enhanced traceability, pathogen testing, and cold-chain verification requirements, eroding profit margins and restricting market access^58,59^. These vulnerabilities would be shaped not only by political boundaries but by regional trade interdependencies, shared ecological characteristics, and infrastructure capacity, with rural and remote communities—often characterized by weaker distribution systems and fewer redundancies—being particularly at risk.

Consumer behavior would also diverge sharply from patterns seen during COVID-19. Even products and producers not implicated in contamination events often suffer spillover reputational damage, amplifying economic losses, as documented in previous outbreaks^54,55^. Given the psychological link between food and health, such demand shocks might persist longer than the mobility-related disruptions of COVID-19. Supply chains would also face distinct challenges. At the same time, COVID-19 demonstrated that shifts in food demand are mediated by the structural divide between food consumed at home and food supplied to restaurants and institutions. During lockdowns, this divide contributed to temporary increases in demand for local and regional producers and alternative food channels, including farmers’ markets and home food production and preservation. These shifts were uneven, context-specific, and largely reversible; many small and medium enterprises, particularly in the food service sector, experienced sustained losses and, in numerous cases, were unable to reopen once restrictions were lifted^13,14^.

Supply chains would also face distinct challenges. While COVID-19 strained logistics due to labor shortages and container imbalances^60^, a foodborne pandemic would necessitate stricter sanitary border controls, increasing clearance times, compliance burdens, and spoilage risks. Regions and countries lacking robust infrastructure resilient distribution capacity, or digitized traceability systems would struggle to meet these requirements, exacerbating inequalities in global and regional food trade.

Overall, whereas COVID-19’s airborne nature disrupted food-related trade broadly but often transiently, a foodborne pandemic would likely produce more targeted, persistent and trust-driven disruptions, with uneven spatial impacts shaped by regional integration, infrastructure conditions, and social vulnerability. These considerations underscore the importance of investing in food safety governance, digital traceability, and international cooperation to mitigate trade shocks and strengthen agrifood system resilience.

### Recommended actions

A variety of actions can strengthen and build resiliency in food chains and food control systems to protect against contamination with pathogens of pandemic potential, and to ensure those supply chains are equipped to withstand and recover from pandemic-related disruptions and guarantee safe, equitable and continuous access to food. These actions are detailed in Table 2, and are equally applicable in both food-exporting economies, where strengthening hygienic production practices, laboratory testing and surveillance capacity from farm to processing can reduce pathogen burden in food products, and import-dependent economies where such measures can ensure product safety on arrival, and as such will enhance global food safety and food security. In addition, investments in resilient food distribution and cold-chain infrastructure are essential to maintain the safety, integrity, and accessibility of food during public health emergencies, particularly for perishable products and for rural and remote communities^2,13,15^. Given the complexity of interactions between human, animal and environmental factors across all levels of the food chain, multisectoral One Health approaches must guide activities to strengthen these systems as part of pandemic preparedness and response plans. Institutionalized coordination mechanisms and multisectoral capacity-building efforts across the public and animal health, agriculture, trade, and other relevant sectors (for example, border security) will provide the necessary foundation to ensure that food safety is fully integrated into pandemic preparedness planning, response, and recovery mechanisms. These efforts will ensure a surge-ready workforce capable of early detection, timely response and risk management of foodborne outbreaks and emergencies^61^.

**Table 2:** Recommendations for pandemic preparedness in food control systems

Thematic area	Recommendation	Expected benefit	Specific activities	Example
Integrated Food Safety and Pandemic Planning	Institutionalize coordination across sectors (food safety, human and animal health, environment, trade, border security, etc.) incorporating food safety into preparedness planning	Ensure food safety is integrated into pandemic preparedness and response	-Support development or enhancement of existing Multisectoral Coordination Mechanisms such as One Health platforms linking human and animal health, environment, agriculture, trade, and emergency services to address pandemic preparedness and response (PPR) and foodborne and zoonotic risks and emergencies -Establish a framework and guidelines for multisectoral communications, data sharing, analysis, and dissemination among human health, animal health, and food industry stakeholders during foodborne and zoonotic health emergencies. -Facilitate regular meetings between human health, animal health, and food industry stakeholders to support coordinated responses to potential foodborne threats. -Strengthen cross-border coordination on PPR and foodborne and zoonotic pathogens control by promoting collaboration with neighboring countries to harmonize quarantine and testing protocols for animal and food imports and exports and share best practices for border biosecurity. -Develop and implement mechanisms for real-time cross-border early warning and information sharing on emerging threats related to foodborne pathogens, including leveraging the International Food Safety Authorities Network (INFOSAN)	The Singapore One Health framework includes the Communicable Diseases Agency (CDA), National Environment Agency, National Parks Board (Animal and Veterinary Service), National Water Agency, and Singapore Food Agency, which collaborate on multisectoral initiatives. Strategic direction, priority setting and coordination and intersectoral collaboration to successfully implement One Health action plans are provided by the Singapore One Health Co-ordinating Committee (OHCC), One Health Working Group, and One Health Office^62^.
Surveillance and early warning	Implement coordinated multisectoral surveillance integrating human, animal, and food safety data	Enable early detection, real-time monitoring and control of foodborne and potential pandemic risks	-Develop national strategy for coordinated multisectoral, real-time monitoring of foodborne and zoonotic diseases and food supply chains -Promote and utilize digital tools and platforms to enable coordinated surveillance and rapid data sharing between stakeholders under the One Health approach to detect, trace, and manage foodborne and zoonotic risks and food safety and supply chain monitoring. -Establish monitoring protocols and implement targeted surveillance at critical points in the food supply chain, including production, processing, and distribution stages for early foodborne and zoonotic disease and AMR detection	- Sudan's 2019 Rift Valley Fever outbreak response demonstrated effective and timely multisectoral coordination and collaboration between the federal Ministries of Health and Animal Resources and other partner ministries and international organizations^63^. -The Canadian Integrated Program for Antimicrobial Resistance Surveillance (CIPARS) integrates and analyzes human medical and agriculture antimicrobial use and resistance data to inform national policy development in collaboration with federal, provincial and territorial, private sector, and international partners^64^.
Laboratory testing capacity	Enhance diagnostic capabilities and integrate lab data into surveillance platforms	Support timely and accurate food safety testing, surveillance and crisis response	- Conduct baseline country capacity analysis using WHO food safety strategy mapping tool -Develop and implement standardized testing protocols aligned with international best practices and train laboratory personnel in modern diagnostic techniques and surveillance methodologies for foodborne and zoonotic pathogens and AMR -Create standard operating procedures (SOPs) for specimen referral and transport between food safety monitoring sites and diagnostic laboratories to support timely and accurate food safety testing. -Use digital technologies to incorporate laboratory testing results into surveillance and food safety and biosafety monitoring platforms.	Singapore’s use of integrated laboratory information management systems (LIMS) helped coordinate public health and lab testing during SARS^65^.
Workforce capacity	Train multisectoral rapid response teams and promote gender-responsive recruitment	Strengthen capacity for outbreak response and inclusive community engagement	- Perform a comprehensive PPR Workforce Capacity gap analysis using FAO and WHO tools across food safety, veterinary, and public health, and other relevant sectors to identify existing shortages, skill gaps, and training needs especially related to food contamination events. -Develop multisectoral rapid response team (RRT) roster templates and standard operating procedures (SOPs) for responses to zoonotic and foodborne disease events and AMR detection within the food supply chain. - Create tailored training modules considering gender-specific challenges and opportunities - Implement targeted training for public health and animal health/veterinary teams, food industry personnel, regulatory bodies, and emergency response teams, based on Codex Alimentarius and other international standards - Introduce interdisciplinary university courses on food safety and emergency preparedness (e.g., within public health, veterinary, or food science faculties)	-The Thai Field epidemiology training program (FETP) and Field epidemiology training program for veterinarians (FETPV) have been a model for decades for sustainable, One Health joint capacity building and training of human, animal and other related health sciences professionals supporting effective detection, prevention and control of major outbreaks of infectious disease and other health threats, and has expanded to train staff from and implemented in other countries in Asia^66^. -Nigeria Centre for Disease Control implemented a One Health emergency response to the 2019 Lassa fever outbreak, with over 5000 cases in states across the country, and deploying multi-sectoral One Health RRTs to provide onsite technical support in the most heavily affected states, operationalizing One Health approaches to control the epidemic and demonstrating the added value of environmental track training in FETP programs^67^.
Regulatory frameworks and policies	Modernize legal frameworks to support resilient agrifood systems and biosecurity measures	Reduce zoonotic spillover risk and improve agrifood system resilience	-Conduct assessment of existing legal frameworks, regulations and policies governing phytosanitary and sanitary animal production practices -Develop legislation, regulations and policies for biosecurity, phytosanitary and sanitary animal production practices and structural and operational controls to reduce the risk of zoonotic spillover and AMR introduction into agrifood systems. - Update food safety laws and regulations to include emergency inspection, traceability, and remote audit provisions - Develop procedures and guidance for managing food safety risks and emergency responses in pandemic settings. - Include agrifood system emergency protocols in national legislation	Following the West Africa Ebola epidemic, Sierra Leone implemented its National Action Plan for Health Security (NAPHS), 2018–2021 to recover and protect against future epidemics, with a whole-of-government approach led by the launch of the NAPHS by the President of Sierra Leone in 2019, multisectoral ministerial-level leadership, strong technical engagement from government, stakeholders and partners, and multi-sectoral coordination led by the International Health Regulation (IHR) National Focal Point and the One Health Secretariat. The Joint Expert Evaluation (JEE) self-assessment scores improved from 44% in 2018 to 57% in 2020 in the human health sector, and from 32% in 2018 to 52% in 2020 in the animal health sector, and the number of indicators with demonstrated capacity increased from 12% in 2018 to 33% in 2020 for human health and from 4% in 2018 to 26% in 2020 for animal health^68^.

## Conclusions

Until food chains and food safety systems are strengthened and fully integrated into preparedness and response planning, their susceptibility to contamination with pathogens with pandemic potential and to pandemic-related disruptions will remain an Achilles’ heel for global health security and food safety and security. Approaches that integrate food safety controls into national plans for pandemic prevention, detection, response and recovery, and that build resiliency in food safety systems and supply chains, can ensure countries are better equipped to withstand pandemic-related disruptions, whether caused by foodborne pathogens or other infectious agents. Such approaches strengthen preparedness capacities across surveillance, laboratory systems, governance, and workforce readiness, and their effectiveness can be reflected in improved performance against established international preparedness and capacity assessment frameworks. At the same time, strengthening food safety systems alone cannot guarantee food security for all populations. Even where food safety governance functions effectively, broader challenges continue to shape food security outcomes. Food safety should therefore be understood as a necessary but not sufficient component of food security, requiring integration with wider agrifood system, social protection, and development policies. These investments not only safeguard public health and ensure continuous access to safe food for all populations but also have demonstrated ability to protect economies and lives and improve livelihoods.

## Supplementary information

Supplemental Table 1
