# Supplementary material for: Building resilient food safety systems through a One Health approach to prepare for the next pandemic
Source: NPJ Sci Food. 2026 Jul 1;10:209. doi: 10.1038/s41538-026-00798-4 (PMC13323731; doi:10.1038/s41538-026-00798-4)
Supplement: Supplementary file 1 — Supplemental Table 1 [file 41538_2026_798_MOESM1_ESM.docx]

**Supplemental Table 1: Foodborne and Food Systems Risk for 2024 WHO R&D Blueprint for Epidemics List of Pathogen Families and Pathogens of High Risk for a Public Health Emergency of Concern**

| **Pathogen name**^[[1]](#footnote-1)^ | **Common name** | **Family** | **Potential to cause human infections**^[[2]](#footnote-2)^**:** | | | **Potential for sustained human-to-human or community transmission**^[[3]](#footnote-3)^ |
| --- | --- | --- | --- | --- | --- | --- |
|  |  |  | **Through consumption of infected or contaminated foods** | **Through contact or handling, slaughter or butchering of infected animals, or meat & animal products in LAM & abattoirs** | **Through contact or handling of infected or contaminated food packaging or food (not infected meat)** |  |
| Mammarenavirus lassaense | Lassa Fever virus | Arenaviridae | Demonstrated occurrence | Does not occur or no supporting evidence | Demonstrated occurrence | Limited (e.g., nosocomial, close contacts) |
| Mammarenavirus juninense | Argentine Hemorrhagic Fever virus | Arenaviridae | Plausible or evidence of potential to occur | Does not occur or no supporting evidence | Demonstrated occurrence | Limited (e.g., nosocomial, close contacts) |
| Mammarenavirus lujoense | Lujo virus | Arenaviridae | Does not occur or no supporting evidence | Does not occur or no supporting evidence | Plausible or evidence of potential to occur | Limited (e.g., nosocomial, close contacts) |
| ***Vibrio cholerae* serogroup O139** | ***Vibrio cholerae* serogroup O139** | Bacteria | **Demonstrated occurrence** | **Does not occur or no supporting evidence** | **Does not occur or no supporting evidence** | **Fecal-oral food or waterborne community transmission** |
| ***Yersinia Pestis*** | ***Yersinia Pestis*** | Bacteria | **Demonstrated occurrence** | **Demonstrated occurrence** | **Does not occur or no supporting evidence** | **Demonstrated sustained human-to-human transmission** |
| ***Shigella dysenteriae* serotype 1** | ***Shigella dysenteriae* serotype 1** | Bacteria | **Demonstrated occurrence** | **Does not occur or no supporting evidence** | **Plausible or evidence of potential to occur** | **Fecal-oral food or waterborne community transmission** |
| ***Salmonella enterica* non-typhoidal serovars** | ***Salmonella enterica* non-typhoidal serovars** | Bacteria | **Demonstrated occurrence** | **Does not occur or no supporting evidence** | **Demonstrated occurrence** | **Fecal-oral food or waterborne community transmission** |
| *Klebsiella pneumoniae* | *Klebsiella pneumoniae* | Bacteria | Demonstrated occurrence | Does not occur or no supporting evidence | Plausible or evidence of potential to occur | Limited (e.g., nosocomial, close contacts) |
| **Coronaviridae Subgenus Merbecovirus** | **MERS-CoV** | Coronaviridae | **Plausible or evidence of potential to occur** | **Demonstrated occurrence** | **Does not occur or no supporting evidence** | **Demonstrated sustained human-to-human transmission** |
| **Coronaviridae Subgenus Sarbecovirus** | **SARS-CoV-1** | Coronaviridae | **Does not occur or no supporting evidence** | **Demonstrated occurrence** | **Does not occur or no supporting evidence** | **Demonstrated sustained human-to-human transmission** |
| **Coronaviridae Subgenus Sarbecovirus** | **SARS-CoV-2** | Coronaviridae | **Does not occur or no supporting evidence** | **Plausible or evidence of potential to occur** | **Demonstrated occurrence** | **Demonstrated sustained human-to-human transmission** |
| Orthoebolavirus zairense | Ebola virus | Filoviridae | Demonstrated occurrence | Demonstrated occurrence | Does not occur or no supporting evidence | Limited (e.g., nosocomial, close contacts) |
| Orthoebolavirus sudanense | Sudan virus | Filoviridae | Plausible or evidence of potential to occur | Plausible or evidence of potential to occur | Does not occur or no supporting evidence | Limited (e.g., nosocomial, close contacts) |
| Orthomarburgvirus marburgense | Marburg virus | Filoviridae | Does not occur or no supporting evidence | Plausible or evidence of potential to occur | Does not occur or no supporting evidence | Limited (e.g., nosocomial, close contacts) |
| Orthoflavivirus zikaense | Zika virus | Flaviviridae | Does not occur or no supporting evidence | Does not occur or no supporting evidence | Does not occur or no supporting evidence | Intimate exposure (e.g., blood exposure or transfusion, organ transplant, sexual contact) |
| Orthoflavivirus denguei | Dengue virus | Flaviviridae | Does not occur or no supporting evidence | Does not occur or no supporting evidence | Does not occur or no supporting evidence | Intimate exposure (e.g., blood exposure or transfusion, organ transplant, sexual contact) |
| Orthoflavivirus flavi | Yellow Fever virus | Flaviviridae | Does not occur or no supporting evidence | Does not occur or no supporting evidence | Does not occur or no supporting evidence | Intimate exposure (e.g., blood exposure or transfusion, organ transplant, sexual contact) |
| Orthoflavivirus encephalitidis | Tick-borne encephalitis virus | Flaviviridae | Demonstrated occurrence | Does not occur or no supporting evidence | Does not occur or no supporting evidence | Intimate exposure (e.g., blood exposure or transfusion, organ transplant, sexual contact) |
| Orthoflavivirus nilense | West Nile Virus | Flaviviridae | Does not occur or no supporting evidence | Demonstrated occurrence | Does not occur or no supporting evidence | Intimate exposure (e.g., blood exposure or transfusion, organ transplant, sexual contact) |
| Orthohantavirus sinnombreense | Sin Nombre virus | Hantaviridae | Does not occur or no supporting evidence | Does not occur or no supporting evidence | Plausible or evidence of potential to occur | Does not occur or no supporting evidence |
| Orthohantavirus hantanense | Hanta virus | Hantaviridae | Does not occur or no supporting evidence | Does not occur or no supporting evidence | Plausible or evidence of potential to occur | Does not occur or no supporting evidence |
| Orthonairovirus haemorrhagiae | Crimean Congo Hemorrhagic Fever virus | Nairoviridae | Plausible or evidence of potential to occur | Demonstrated occurrence | Does not occur or no supporting evidence | Intimate exposure (e.g., blood exposure or transfusion, organ transplant, sexual contact) |
| Alphainfluenzavirus Influenzae H1^[[4]](#footnote-4)^ | Influenza A H1 virus | Orthomyxo- viridae | Does not occur or no supporting evidence | Plausible or evidence of potential to occur | Does not occur or no supporting evidence | Demonstrated sustained human-to-human transmission |
| Alphainfluenzavirus Influenzae H5^4^ | Influenza A H5 virus | Orthomyxo- viridae | Plausible or evidence of potential to occur | Demonstrated occurrence | Does not occur or no supporting evidence | Does not occur or no supporting evidence |
| Henipavirus nipahense | Nipah and henipah viruses | Paramyxoviridae | Demonstrated occurrence | Demonstrated occurrence | Does not occur or no supporting evidence | Limited (e.g., nosocomial, close contacts) |
| Bandavirus dabieense | Severe Fever with Thrombocytopenia Syndrome Virus (SFTSV) | Phenuiviridae | Does not occur or no supporting evidence | Does not occur or no supporting evidence | Does not occur or no supporting evidence | Limited (e.g., nosocomial, close contacts) |
| Phlebovirus riftense | Rift Valley Fever virus | Phenuiviridae | Plausible or evidence of potential to occur | Demonstrated occurrence | Does not occur or no supporting evidence | Does not occur or no supporting evidence |
| Orthopoxvirus variola | Variola (smallpox) virus | Poxviridae | Does not occur or no supporting evidence | Does not occur or no supporting evidence | Does not occur or no supporting evidence | Demonstrated sustained human-to-human transmission |
| Orthopoxvirus vaccinia | Vaccinia (cowpox) virus | Poxviridae | Plausible or evidence of potential to occur | Demonstrated occurrence | Does not occur or no supporting evidence | Does not occur or no supporting evidence |
| Orthopoxvirus monkeypox | mpox virus | Poxviridae | Demonstrated occurrence | Demonstrated occurrence | Does not occur or no supporting evidence | Limited (e.g., nosocomial, close contacts) |
| Alphavirus chikungunya | Chikungunya virus | Togaviridae | Does not occur or no supporting evidence | Does not occur or no supporting evidence | Does not occur or no supporting evidence | Intimate exposure (e.g., blood exposure or transfusion, organ transplant, sexual contact) |
| Alphavirus venezuelan | Venezuelan Equine Encephalitis virus | Togaviridae | Does not occur or no supporting evidence | Does not occur or no supporting evidence | Does not occur or no supporting evidence | Does not occur or no supporting evidence |

Resources:

Potential resources including primary research or investigation reports and review articles for supporting evidence of the potential for each listed pathogen to cause human infection through one of the three food-related exposures, and for epidemic spread through human-to-human transmission or community transmission through fecal contamination of food or water, were identified using the National Library of Medicine PubMed® (https://pubmed.ncbi.nlm.nih.gov/) search engine with the following keyword searches: [((pathogen common name) AND (food)) AND (contamination)], [(pathogen common name) AND (epidemiology)], and [(pathogen common name) AND (review)]. Additional references, webpages and other primary and review resources were identified using similar search strategies on the online Google search engine. Reference abstracts were screened and references and online resources reviewed; key relevant resources and references supporting the assessments are listed below.

| Lassa Fever virus:  National Institute for Communicable Diseases Centre for Emerging Zoonotic and Parasitic Diseases, South Africa. (2022). Lassa fever: Frequently Asked Questions. <https://www.nicd.ac.za/wp-content/uploads/2022/05/Lassa-Fever-FAQ_13-May-2022_FINAL.pdf> Accessed 20 June 2025.  Ter Meulen, J., Lukashevich, I., Sidibe, K., Inapogui, A., Marx, M., Dorlemann, A., Yansane, M.L., Koulemou, K., Chang-Claude, J., Schmitz, H. (1996). Hunting of peridomestic rodents and consumption of their meat as possible risk factors for rodent-to-human transmission of Lassa virus in the Republic of Guinea. *Am J Trop Med Hyg*, 55(6), 661-6. doi: 10.4269/ajtmh.1996.55.661.  World Health Organization. (2024). Lassa fever. <https://www.who.int/news-room/fact-sheets/detail/lassa-fever>. Accessed 20 June 2025. |
| --- |
| Argentine Hemorrhagic Fever virus:  Sultana, M., Nabilah, N., Islam, S.M.S., Uddin, M.I., Nisha, A.S., Snigdha, M.F., Islam, M.Z., Syrmos, N., Afrin, S., Mahedi, M.R.A. (2023). A Narrative Review on Argentine Hemorrhagic Fever: Junin Virus (JUNV). *J Clin Immunol Microbiol*, 4(2), 1-4. http://dx.doi.org/10.46889/JCIM.2023.4202 |
| Lujo virus:  Simulundu, E., Mweene, A.S., Changula, K., Monze, M., Chizema, E., Mwaba, P., Takada, A., Ippolito, G., Kasolo, F., Zumla, A., Bates, M. (2016). Lujo viral hemorrhagic fever: considering diagnostic capacity and preparedness in the wake of recent Ebola and Zika virus outbreaks. *Rev Med Virol*, 26(6), 446-454. doi: 10.1002/rmv.1903. |
| *Vibrio cholerae* serogroup O139:  Appelt, S., Nair, S., Barker, C.R., Jenkins, C., Gatz, J., Rohleder, A.M., Scholz, H.C., Dupke, S. (2025). Import of multidrug-resistant Vibrio cholerae from Ethiopia to Germany and the UK. *Lancet Microbe*, 101179. doi: 10.1016/j.lanmic.2025.101179.  Davis, W., Narra, R., Mintz, E.D. (2018). Cholera. *Curr Epidemiol Rep*, 5(3), 303-315. doi: 10.1007/s40471-018-0162-z.  Forssman, B., Mannes, T., Musto, J., Gottlieb, T., Robertson, G., Natoli, J.D., Shadbolt, C., Biffin, B., Gupta, L. (2007). Vibrio cholerae O1 El Tor cluster in Sydney linked to imported whitebait. *Med J Aust*, 187(6), 345-7. doi: 10.5694/j.1326-5377.2007.tb01278.x.  Johns Hopkins University. (2024). An Updated Estimate on the Burden of Cholera in Endemic Countries. <https://publichealth.jhu.edu/sites/default/files/2025-01/An-Updated-Estimate-on-the-Burden-of-Cholera-in-Endemic-Countries-December-2024.pdf>. Accessed 30 June 2025.  Rabbani, G.H., Greenough 3rd, W.B. (1999) Food as a vehicle of transmission of cholera. J Diarrhoeal Dis Res, 17(1), 1-9.  Teixeira, J.S., Dussault, F., Hoover, E., Shay, J.A., Weedmark, K., Banerjee, S.K.(2022). Draft Genomes of 92 *Vibrio* Isolates from Warm-Water Shrimps Imported into Canada between 2009 and 2019. *Microbiol Resour Announc*, 11(12), e0075022. doi: 10.1128/mra.00750-22. |
| *Yersinia pestis*:  Leslie, T., Whitehouse, C.A., Yingst, S., Baldwin, C., Kakar, F., Mofleh, J., Hami, A.S., Mustafa, L., Omar, F., Ayazi, E., Rossi, C., Noormal, B., Ziar, N., Kakar, R. (2011). Outbreak of gastroenteritis caused by Yersinia pestis in Afghanistan. *Epidemiol Infect*, 139(5), 728-35. doi: 10.1017/S0950268810001792.  Mead, P.S. (2011). Plague. Tropical Infectious Diseases: Principles, Pathogens and Practice, 276–83. doi: 10.1016/B978-0-7020-3935-5.00041-0. |
| *Shigella dysenteriae* serotype 1:  Aslam, A., Hashmi, M.F., Okafor, C.N. (2025) .Shigellosis. In *StatPearls*. StatPearls Publishing. <https://www.ncbi.nlm.nih.gov/books/NBK482337/>  Crotta, M., Prakashbabu, B.C., Holt, H., Swift, B., Pedada, V.C., Shaik, T.B., Kaur, P., Bedi, J.S., Tumati, S.R., Guitian, J. (2022). Microbiological risk ranking of foodborne pathogens and food products in scarce-data settings. *Food Control*, 141, 109152.  Hale, T.L., Keusch, G.T. (1996). Chapter 22: Shigella. In: Medical Microbiology. 4th ed. Baron S, editor. Galveston (TX): University of Texas Medical Branch at Galveston. |
| *Salmonella enterica* non-typhoidal serovars (NTS):  Gal-Mor, O., Boyle, E.C., Grassl, G.A. (2014). Same species, different diseases: how and why typhoidal and non-typhoidal Salmonella enterica serovars differ. *Front Microbiol*, 4(5), 391. doi: 10.3389/fmicb.2014.00391.  Kumar, G., Kumar, S., Jangid, H., Dutta, J., Shidiki, A. (2025). The rise of non-typhoidal *Salmonella*: an emerging global public health concern. *Front Microbiol*, 16,1524287. doi: 10.3389/fmicb.2025.1524287.  Lamichhane, B., Mawad, A. M. M., Saleh, M., Kelley, W. G., Harrington, P. J., II, Lovestad, C. W., Amezcua, J., Sarhan, M. M., El Zowalaty, M. E., Ramadan, H., Morgan, M., & Helmy, Y. A. (2024). Salmonellosis: An Overview of Epidemiology, Pathogenesis, and Innovative Approaches to Mitigate the Antimicrobial Resistant Infections. *Antibiotics*, 13(1), 76. <https://doi.org/10.3390/antibiotics13010076> |
| *Klebsiella pneumoniae*:  Hartantyo, S.H.P., Chau, M.L., Koh, T.H., Yap, M., Yi, T., Cao, D.Y.H., Gutiérrez, R.A., Ng ,L.C. (2020). Foodborne Klebsiella pneumoniae: Virulence Potential, Antibiotic Resistance, and Risks to Food Safety. *J Food Prot*, 83(7), 1096-1103. doi: 10.4315/JFP-19-520.  Riwu, K.H.P., Effendi, M.H., Rantam, F.A., Khairullah, A.R., Widodo, A. (2022). A review: Virulence factors of Klebsiella pneumonia as emerging infection on the food chain. *Vet World*, 15(9), 2172–2179. doi: 10.14202/vetworld.2022.2172-2179  Davis, G.S., Price, L.B. (2016). Recent Research Examining Links Among Klebsiella pneumoniae from Food, Food Animals, and Human Extraintestinal Infections. *Curr Environ Health Rep*, 3(2), 128-35. doi: 10.1007/s40572-016-0089-9. |
| MERS-CoV:  Han, H.J., Yu, H., Yu, X.J. (2016). Evidence for zoonotic origins of Middle East respiratory syndrome coronavirus. *J Gen Virol*, 97(2), 274-280. doi: 10.1099/jgv.0.000342.  Mohamed, M.-Y.I., Lakshmi, G.B., Sodagari, H., Habib, I. (2024). A One Health Perspective on Camel Meat Hygiene and Zoonoses: Insights from a Decade of Research in the Middle East. *Vet Sci*, 11, 344. https://doi.org/10.3390/vetsci11080344.  van Doremalen, N., Bushmaker, T., Karesh, W.B., Munster, V.J. (2014). Stability of Middle East respiratory syndrome coronavirus in milk. *Emerg Infect Dis*, 20(7), 1263-4. doi: 10.3201/eid2007.140500. |
| SARS-CoV-1:  Bell, D., Roberton, S., Hunter, P.R. (2004). Animal origins of SARS coronavirus: possible links with the international trade in small carnivores. *Philos Trans R Soc Lond B Biol Sci*, 359(1447), 1107-14. doi: 10.1098/rstb.2004.1492  Wang, M., Yan, M., Xu, H., Liang, W., Kan, B., Zheng, B., Chen, H., Zheng, H., Xu, Y., Zhang, E., Wang, H., Ye, J., Li, G., Li, M., Cui, Z., Liu, YF., Guo, R.T., Liu, X.N., Zhan, L.H., Zhou, D.H., Zhao, A., Hai, R., Yu, D., Guan, Y., Xu, J. (2005). SARS-CoV infection in a restaurant from palm civet. *Emerg Infect Dis*, 11(12), 1860-5. doi: 10.3201/eid1112.041293.  Xu, R.H., He, J.F., Evans, M.R., Peng, G.W., Field, H.E., Yu, D.W., Lee, C.K., Luo, H.M., Lin, W.S., Lin, P., Li, L.H., Liang, W.J., Lin, J.Y., Schnur, A. (2004). Epidemiologic clues to SARS origin in China. *Emerg Infect Dis*, 10(6), 1030-7. doi: 10.3201/eid1006.030852. |
| SARS-CoV-2:  Chen, C., Feng, Y., Chen, Z., Xia, Y., Zhao, X., Wang, J., Nie, K., Niu, P., Han, J., & Xu, W. (2022). SARS-CoV-2 cold-chain transmission: Characteristics, risks, and strategies. *Journal of medical virology*, *94*(8), 3540–3547. <https://doi.org/10.1002/jmv.27750>  Crits-Christoph A, Levy JI, Pekar JE, Goldstein SA, Singh R, Hensel Z, Gangavarapu K, Rogers MB, Moshiri N, Garry RF, Holmes EC, Koopmans MPG, Lemey P, Peacock TP, Popescu S, Rambaut A, Robertson DL, Suchard MA, Wertheim JO, Rasmussen AL, Andersen KG, Worobey M, Débarre F. (2024) Genetic tracing of market wildlife and viruses at the epicenter of the COVID-19 pandemic. *Cell*, 187(19), 5468-5482.e11. doi: 10.1016/j.cell.2024.08.010.  Ma, H., Wang, Z., Zhao, X., Han, J., Zhang, Y., Wang, H., Chen, C., Wang, J., Jiang, F., Lei, J., Song, J., Jiang, S., Zhu, S., Liu, H., Wang, D., Meng, Y., Mao, N., Wang, Y., Zhu, Z., Chen, Z., … Xu, W. (2021). Long Distance Transmission of SARS-CoV-2 from Contaminated Cold Chain Products to Humans - Qingdao City, Shandong Province, China, September 2020. *China CDC weekly*, *3*(30), 637–644. https://doi.org/10.46234/ccdcw2021.164. |
| Ebola virus:  Food and Agriculture Organization of the United Nations. (2018). Addressing Zaire Ebolavirus (EBOV) Outbreaks: Qualitative entry and exposure assessment update. Rome. https://openknowledge.fao.org/server/api/core/bitstreams/ff4ed400-d3c9-427b-809f-125ab6a550e1/content  Mann, E., Streng, S., Bergeron, J., Kircher, A. (2015). A Review of the Role of Food and the Food System in the Transmission and Spread of Ebolavirus. *PLoS Negl Trop Dis*, 9(12), e0004160. doi:10.1371/journal.pntd.0004160  Osterholm, M.T., Moore, K.A., Kelley, N.S., Brosseau, L.M., Wong, G., Murphy, F.A., Peters, C.J., LeDuc, J.W., Russell, P.K., Van Herp, M., Kapetshi, J., Muyembe, J.J., Ilunga, B.K., Strong, J.E., Grolla, A., Wolz, A., Kargbo, B., Kargbo, D.K., Sanders, D.A., Kobinger, G.P. (2015). Transmission of Ebola viruses: what we know and what we do not know. *mBio*, 6(2), e00137. doi: 10.1128/mBio.00137-15. |
| Sudan virus:  Atherstone, C., Smith, E., Ochungo, P., Roesel, K., Grace, D. (2017). Assessing the Potential Role of Pigs in the Epidemiology of Ebola Virus in Uganda. *Transbound Emerg Dis*, 64(2), 333-343. doi: 10.1111/tbed.12394.  Food and Agriculture Organization of the United Nations. (2018). Addressing Zaire Ebolavirus (EBOV) Outbreaks: Qualitative entry and exposure assessment update. Rome. |
| Marburg virus:  Hunter, N., & Rathish, B. (2025). Marburg Virus Disease. In *StatPearls*. StatPearls Publishing. <https://www.ncbi.nlm.nih.gov/books/NBK578176/>  World Health Organization. (2025). Marburg virus disease. <https://www.who.int/news-room/fact-sheets/detail/marburg-virus-disease>. Accessed 30 June 2025. |
| Zika virus:  Centers for Disease Control and Prevention. (2025). Transmission of Zika Virus. https://www.cdc.gov/zika/php/transmission/index.html  World Health Organization. (2022). Zika virus. <https://www.who.int/news-room/fact-sheets/detail/zika-virus> |
| Dengue virus:  Murray, N.E., Quam, M.B., Wilder-Smith, A. (2013). Epidemiology of dengue: past, present and future prospects. *Clin Epidemiol*, 20(5), 299-309. doi: 10.2147/CLEP.S34440. |
| *Yellow Fever virus*:  Gianchecchi, E., Cianchi, V., Torelli, A., Montomoli, E. (2022). Yellow Fever: Origin, Epidemiology, Preventive Strategies and Future Prospects. *Vaccines* (Basel), 10(3), 372. doi: 10.3390/vaccines10030372.  World Health Organization. 2025. Yellow fever. <https://www.who.int/health-topics/yellow-fever#tab=tab_1>. Accessed 30 June 2025. |
| Tick-borne encephalitis virus:  Buczek, A.M., Buczek,W., Buczek, A., Wysokinska-Miszczuk, J. (2022). Food-Borne Transmission of Tick-Borne Encephalitis Virus—Spread, Consequences, and Prophylaxis. *Int J Environ Res Public Health*, 19, 1812. https://doi.org/10.3390/ijerph19031812.  Tomassone, L., Martello, E., Mannelli, A., Vicentini, A., Gossner, C.M., Leonardi-Bee, J. (2025). A Systematic Review on the Prevalence of Tick-Borne Encephalitis Virus in Milk and Milk Products in Europe. *Zoonoses Public Health*, 72(3), 248-258. doi: 10.1111/zph.13216. |
| West Nile Virus:  Kramer, L.D., Styer, L.M., Ebel, G.D. (2008). A global perspective on the epidemiology of West Nile virus. *Annu Rev Entomol*. 2008;53:61-81. doi: 10.1146/annurev.ento.53.103106.093258.  Spickler, AR. 2023. West Nile Virus Infection. Retrieved from http://www.cfsph.iastate.edu/DiseaseInfo/factsheets.php. Accessed 29 Jul 2025. |
| Sin Nombre virus:  Richardson, K.S., Kuenzi, A., Douglass, R.J., Hart, J., Carver, S. (2013). Human exposure to particulate matter potentially contaminated with sin nombre virus. *Ecohealth*, 10(2), 159-65. doi: 10.1007/s10393-013-0830-x.  National Emerging Special Pathogens Training & Education Center. (2024). Understanding Hantavirus Pulmonary Syndrome: Risks, Symptoms, and Prevention. https://netec.org/2024/03/11/understanding-hantavirus-pulmonary-syndrome-risks-symptoms-and-prevention/. |
| Hanta virus:  Canadian Centre for Occupational Health and Safety. (2022). Disease, Disorders and Injuries: Hantavirus. 27 Apr 2022. <https://www.ccohs.ca/oshanswers/diseases/hantavir.html>. Accessed 20 Jul 2025  Toledo, J., Haby, M.M., Reveiz, L., Sosa Leon, L., Angerami, R., Aldighieri, S. (2022). Evidence for Human-to-Human Transmission of Hantavirus: A Systematic Review. *J Infect Dis*, 226(8), 1362-1371. doi: 10.1093/infdis/jiab461. |
| Crimean Congo Hemorrhagic Fever virus:  Fazlalipour, M., Baniasadi, V., Mirghiasi, S.M., Jalali, T., Khakifirouz, S., Azad-Manjiri, S., Mahmoodi, V., Naderi, H.R., Zarandi, R., Salehi-Vaziri, M. (2016). Crimean-Congo Hemorrhagic Fever Due to Consumption of Raw Meat: Case Reports From East-North of Iran. *Jpn J Infect Dis*, 69(3), 270-1. doi: 10.7883/yoken.JJID.2015.498.  Metanat, M., Sharifi-Mood, B., Fathollahzadeh, N., Kahka, L.R., Bahremand, F., Sharifi, R. (2018). Report of a Family with Crimean-Congo Hemorrhagic Fever Following Contact with Frozen Meat: A Case Seires Study. *Arch Clin Infect Dis*, 13(1), e14933. doi: 10.5812/archcid.14933  Sharifi-Mood, B., Metanat, M., Hashemi-Shahri, S.M., Mardani, M., Hashemi, S.A., Jahani, F.F. (2011). Crimean-Congo hemorrhagic fever following consumption of uncooked liver: Case series study. *Iran J Clin Infect Dis*, 6(3), 128-130.  World Health Organization. (2025). Crimean-Congo haemorrhagic fever. <https://www.who.int/news-room/fact-sheets/detail/crimean-congo-haemorrhagic-fever>. Accessed 29 June 2025. |
| Influenza A H1 virus (influenza prototype pathogen):  Borkenhagen, L.K., Wang, G.L., Simmons, R.A., Bi, Z.Q., Lu, B., Wang, X.J., Wang, C.X., Chen, S.H., Song, S.X., Li, M., Zhao, T., Wu, M.N., Park, L.P., Cao, W.C., Ma, M.J., Gray, G.C. (2020). High Risk of Influenza Virus Infection Among Swine Workers: Examining a Dynamic Cohort in China. *Clin Infect Dis*, 71(3), 622-629. doi: 10.1093/cid/ciz865.  Choi, M.J., Torremorell, M., Bender, J.B., Smith, K., Boxrud, D., Ertl, J.R., Yang, M., Suwannakarn, K., Her, D., Nguyen, J., Uyeki, T.M., Levine, M., Lindstrom, S., Katz, J.M., Jhung, M., Vetter, S., Wong, K.K., Sreevatsan, S., Lynfield, R. (2015). Live Animal Markets in Minnesota: A Potential Source for Emergence of Novel Influenza A Viruses and Interspecies Transmission. *Clin Infect Dis*, 61(9), 1355-62. doi: 10.1093/cid/civ618.  European Food Safety Authority (EFSA). (2010). Scientific Opinion of the EFSA Panel on Biological Hazards (BIOHAZ): statement on food safety considerations of novel H1N1 influenza virus infections in humans. *EFSA J*. 8, 1629.  Jilani, T.N., Jamil, R.T., Nguyen, A.D., Siddiqui, A.H. (2024). H1N1 Influenza. In *StatPearls*. StatPearls Publishing. <https://www.ncbi.nlm.nih.gov/books/NBK513241/>  Le Sage, V., Rockey, N.C., French, A.J., McBride, R., McCarthy, K.R., Rigatti, L.H., Shephard, M.J., Jones, J.E., Walter, S.G., Doyle, J.D., Xu, L., Barbeau, D.J., Wang, S., Frizzell, S.A., Myerburg, M.M., Paulson, J.C., McElroy, A.K., Anderson, T.K., Vincent Baker, A.L., Lakdawala, S.S. (2024). Potential pandemic risk of circulating swine H1N2 influenza viruses. *Nat Commun*, 15(1), 5025. doi: 10.1038/s41467-024-49117-z. |
| Influenza A H5 virus (influenza prototype pathogen):  Beigel, J.H., Farrar, J., Han, A.M., Hayden, F.G., Hyer, R., de Jong, M.D., Lochindarat, S., Nguyen, T.K., Nguyen, T.H., Tran, T.H., Nicoll, A., Touch, S., Yuen, K.Y., Writing Committee of the World Health Organization (WHO) Consultation on Human Influenza A/H5. (2005). Avian influenza A (H5N1) infection in humans. *N Engl J Med*, 353(13), 1374-85. doi: 10.1056/NEJMra052211.  Bellido-Martín, B., Rijnink, W.F., Iervolino, M., Kuiken, T., Richard, M., Fouchier, R.A.M. (2025). Evolution, spread and impact of highly pathogenic H5 avian influenza A viruses. *Nat Rev Microbiol*, https://doi.org/10.1038/s41579-025-01189-4  FAO, WHO, WOAH. (2025). Updated joint FAO/WHO/WOAH public health assessment of recent influenza A(H5) virus events in animals and people. <https://www.who.int/publications/m/item/updated-joint-fao-who-woah-public-health-assessment-of-recent-influenza-a(h5)-virus-events-in-animals-and-people-july2025>. Accessed 20 July 2025.  Lin, T.H., Zhu, X., Wang, S., Zhang, D., McBride, R., Yu, W., Babarinde, S., Paulson, J.C., Wilson, I.A. (2024). A single mutation in bovine influenza H5N1 hemagglutinin switches specificity to human receptors. *Science*, 386(6726), 1128-1134. doi: 10.1126/science.adt0180.  Nooruzzaman, M., de Oliveira, P.S.B., Martin, N.H., Alcaine, S.D., Diel, D.G. (2025). Stability of influenza A H5N1 virus in raw milk cheese. *bioRxiv* 2025.03.13.643009. doi: https://doi.org/10.1101/2025.03.13.643009 |
| Nipah and henipah viruses:  Hughes, J.M., Wilson, M.E., Luby, S.P., Gurley, E.S., Hossain, M.J. (2009). Transmission of Human Infection with Nipah Virus. *Clin Infect Dis*, 49(11), 1743–1748. https://doi.org/10.1086/647951.  Rahman, M.A., Hossain, M.J., Sultana, S., Homaira, N., Khan, S.U., Rahman, M., Gurley, E.S., Rollin, P.E., Lo, M.K., Comer, J.A., Lowe, L., Rota, P.A., Ksiazek, T.G., Kenah, E., Sharker, Y., Luby, S.P. (2012). Date palm sap linked to Nipah virus outbreak in Bangladesh, 2008. *Vector Borne Zoonotic Dis*, 12(1), 65-72. doi: 10.1089/vbz.2011.0656.  Singh, R.K., Dhama, K., Chakraborty, S., Tiwari, R., Natesan, S., Khandia, R., Munjal, A., Vora, K.S., Latheef, S.K., Karthik, K., Singh, M.Y., Singh, R., Chaicumpa, W., Mourya, D.T. (2019). Nipah virus: epidemiology, pathology, immunobiology and advances in diagnosis, vaccine designing and control strategies - a comprehensive review. *Vet Q*, 39(1), 26-55. doi: 10.1080/01652176.2019.1580827. |
| Severe Fever with Thrombocytopenia Syndrome Virus (SFTSV):  Kim, E-H., Park, S-J. (2023). Emerging Tick-Borne Dabie bandavirus: Virology, Epidemiology, and Prevention. *Microorganisms*, 11, 2309. https://doi.org/10.3390/microorganisms11092309. |
| Rift Valley Fever virus:  Anyangu, A.S., Gould, L.H., Sharif, S.K., Nguku, P.M., Omolo, J.O., Mutonga, D., Rao, C.Y., Lederman, E.R., Schnabel, D., Paweska, J.T., Katz, M., Hightower, A., Njenga, M.K., Feikin, D.R., Breiman, R.F. (2010). Risk factors for severe Rift Valley fever infection in Kenya, 2007. *Am J Trop Med Hyg*, 83(2 Suppl), 14-21. doi: 10.4269/ajtmh.2010.09-0293.  Grossi-Soyster, E.N., Lee, J., King, C.H., LaBeaud, A.D. (2019). The influence of raw milk exposures on Rift Valley fever virus transmission. *PLoS Negl Trop Dis*, 13(3), e0007258. https://doi.org/10.1371/journal.pntd.0007258.  Nguku, P.M., Sharif, S.K., Mutonga, D., Amwayi, S., Omolo, J., Mohammed, O., Farnon, E.C., Gould, L.H., Lederman, E., Rao, C., Sang, R., Schnabel, D., Feikin, D.R., Hightower, A., Njenga, M.K., Breiman, R.F. (2010). An investigation of a major outbreak of Rift Valley fever in Kenya: 2006-2007. *Am J Trop Med Hyg*, 83(2 Suppl), 5-13. doi: 10.4269/ajtmh.2010.09-0288.  World Health Organization. (2024). Rift Valley fever. <https://www.who.int/news-room/fact-sheets/detail/rift-valley-fever>. Accessed 29 June 2025. |
| Variola (smallpox) virus:  Milton, D.K. (2012). What was the primary mode of smallpox transmission? Implications for biodefense. *Front Cell Infect Microbiol*, 2, 150. doi: 10.3389/fcimb.2012.00150.  Simonsen, K.A., Snowden, J. (2023). Smallpox. In *StatPearls*. StatPearls Publishing. <https://www.ncbi.nlm.nih.gov/books/NBK470418/>. |
| Vaccinia (cowpox) virus:  Costa, G.B., Borges, I.A., Alves, P.A., Miranda, J.B., Luiz, A.P., Ferreira, P.C., Abrahão, J.S., Moreno, E.C., Kroon, E.G., Trindade, G.S. (2015). Alternative Routes of Zoonotic Vaccinia Virus Transmission, Brazil. *Emerg Infect Dis*, 21(12), 2244-6. doi: 10.3201/eid2112.141249.  Oliveira, J.S., Figueiredo, P.O., Costa, G.B., Assis, F.L., Drumond, B.P., da Fonseca, F.G., Nogueira, M.L., Kroon, E.G., Trindade, G.S. (2017). Vaccinia Virus Natural Infections in Brazil: The Good, the Bad, and the Ugly. *Viruses*, 9(11), 340. doi: 10.3390/v9110340. |
| mpox virus:  Alakunle, E., Moens, U., Nchinda, G., Okeke, M.I. (2020). Monkeypox Virus in Nigeria: Infection Biology, Epidemiology, and Evolution. *Viruses,* 12(11), 1257. doi: 10.3390/v12111257.  Chaix, E., Boni, M., Guillier, L., Bertagnoli, S., Mailles, A., Collignon, C., Kooh, P., Ferraris, O., Martin-Latil, S., Manuguerra, J.C., Haddad, N. (2022). Risk of Monkeypox virus (MPXV) transmission through the handling and consumption of food. *Microb Risk Anal*, 22, 100237. doi: 10.1016/j.mran.2022.100237.Reynolds, M. G., Wauquier, N., Li, Y., Satheshkumar, P. S., Kanneh, L. D., Monroe, B., Maikere, J., Saffa, G.,  Gonzalez, J. P., Fair, J., Carroll, D. S., Jambai, A., Dafae, F., Khan, S. H., & Moses, L. M. (2019). Human Monkeypox in Sierra Leone after 44-Year Absence of Reported Cases. *Emerging infectious diseases*, *25*(5), 1023–1025. <https://doi.org/10.3201/eid2505.180832>  Rimoin, A.W., Mulembakani, P.M., Johnston, S.C., Lloyd Smith, J.O., Kisalu, N.K., Kinkela, T.L., Blumberg, S., Thomassen, H.A., Pike, B.L., Fair, J.N., Wolfe, N.D., Shongo, R.L., Graham, B.S., Formenty, P., Okitolonda, E., Hensley, L.E., Meyer, H., Wright, L.L., Muyembe, J.J. (2010). Major increase in human monkeypox incidence 30 years after smallpox vaccination campaigns cease in the Democratic Republic of Congo. *Proc Natl Acad Sci U S A*, 107(37), 16262-7. doi: 10.1073/pnas.1005769107. |
| Chikungunya virus:  Centers for Disease Control and Prevention. (2025). Chikungunya: Causes and How It Spreads.  <https://www.cdc.gov/chikungunya/causes-and-spread/index.html>. Accessed 20 June 2025.  World Health Organization. (2025). Chikungunya. <https://www.who.int/news-room/fact-sheets/detail/chikungunya>. Accessed 20 June 2025. |
| Venezuelan Equine Encephalitis virus:  Aguilar, P.V., Estrada-Franco, J.G., Navarro-Lopez, R., Ferro, C., Haddow, A.D., Weaver, S.C. (2011). Endemic Venezuelan equine encephalitis in the Americas: hidden under the dengue umbrella. *Future Virol*, 6(6), 721-740. PMID: 21765860.  Crosby, B., Crespo, M.E. (2023). Venezuelan Equine Encephalitis. In *StatPearls*. StatPearls Publishing. <https://www.ncbi.nlm.nih.gov/books/NBK559332/>. |

1. Pathogens listed in bold were assessed as having the potential to cause a PHEIC or pandemic following food-related introduction into human populations. Assessment was based on the pathogen having demonstrated occurrence of having caused human infections following either: consumption of infected or contaminated foods; contact with or handling of food animals and animal products including in live animal markets (LAM), slaughter and butchering, or handling of meat and other animal products; or handling contaminated foods or food packaging, AND having potential for epidemic spread through human-to-human transmission or community transmission through fecal contamination of food or water. [↑](#footnote-ref-1)
2. Values based on existing evidence: Demonstrated occurrence; Plausible or evidence of potential to occur; Does not occur or no supporting evidence. [↑](#footnote-ref-2)
3. Values based on existing evidence: Demonstrated human-to-human transmission; Fecal-oral food or waterborne community transmission; Limited (e.g., nosocomial, close contacts); Intimate exposure (e.g., blood exposure or transfusion, organ transplant, sexual contact); Does not occur or no supporting evidence. [↑](#footnote-ref-3)
4. Influenza A H1 and H5 viruses are evaluated as the Prototype Pathogens representing Alphainfleunzavirus influenzae H1, H2, H3, H5, H6, H7, and H10 [↑](#footnote-ref-4)
